# Nutrition for women and children—Are we doing the right things in the right way?

**DOI:** 10.1371/journal.pmed.1002906

**Published:** 2019-08-27

**Authors:** Lars Åke Persson, Kathleen M. Rasmussen, Huixia Yang

**Affiliations:** 1 London School of Hygiene & Tropical Medicine, London, United Kingdom; 2 Ethiopian Public Health Institute, Addis Ababa, Ethiopia; 3 Division of Nutritional Sciences, Cornell University, Ithaca, New York, United States of America; 4 Department of Obstetrics and Gynecology, Peking University First Hospital, Beijing, China

## Abstract

The Guest Editors for the PLOS Medicine Special Issue on Maternal and Child Health & Nutrition discuss the published research in the context of global priorities for women's and children's health.

The recent 2019 report on the State of Food Security and Nutrition in the World paints a picture of some progress, but also highlights alarming developments in certain areas [1]. After a period of decline, hunger is now increasing, and over 2 billion people do not have regular access to safe, nutritious and sufficient food. Hunger has also increased in middle-income countries. Anemia in women continues to be a widespread problem, and there is a gender gap with more women than men being food insecure. One in seven newborns suffered from low birth weight in 2015; there has been no improvement in recent years. The number of children with stunted linear growth has decreased, but this progress is insufficient to meet the 2030 target of halving the total number of stunted children. Currently, almost 150 million children are stunted, and 50 million suffer from wasting.

At the same time, overweight and obesity are increasing in all regions of the world, especially among school-age children and adults. Many low- and middle-income countries suffer a double burden of malnutrition, with maternal and child malnutrition a particular problem, alongside increasing occurrence of adult overweight, insulin resistance, diabetes, and cardiovascular diseases [2]. In the accompanying *PLOS Medicine* Special Issue on Maternal and Child Health & Nutrition, important research contributions are provided from three cross-cutting perspectives: the ongoing social and nutritional transitions in societies, the woman-and-child continuum of health and nutrition care, and that of the Developmental Origins of Health and Disease (DOHaD).

The risk of next-generation overweight and obesity is increased when the mother is obese before conception [3]. Countries with an ongoing rapid nutritional transition, for example Bangladesh, show drastic increases in overweight and obesity in women of reproductive age [4]. This has severe adverse effects for women’s health, pregnancy outcomes, and the long-term health of their offspring. In the Special Issue, a number of scientific contributions on the mother-and-child dyad focus on weight change, overweight, obesity and related conditions and outcomes [5–8]. Two studies address how women could be helped to achieve an appropriate weight before pregnancy, and whether achieving that weight improves outcomes. When women with obesity had bariatric surgery to induce weight loss before pregnancy, Zainab Akhter and colleagues show in their systematic review and meta-analysis that the odds of a number of adverse birth outcomes increased [6]. In a study on the ELFE birth cohort in France, Marion Lecourgillé and colleagues considered the role of weight change in women either above or below the upper end of normal body mass index. In both groups, they found that gestational weight gain mediated the effects of pre-pregnancy weight change on infant birth weight [7].

Breastfeeding is a fundamental early element of infant nutrition. An analysis of data from 49 African countries over the period 2000–2017 showed that an increasing proportion of infants aged under six months were exclusively breastfed, especially in the eastern part of sub-Saharan Africa [9]. There are, however, many countries that are unlikely to reach the WHO global nutrition target of at least 50% of infants exclusively breastfed by 2025, and suboptimal breastfeeding results in about 800,000 child deaths each year [10]. There are several evidence-based strategies, such as the establishment of baby-friendly hospitals and clinics, and peer counseling for exclusive breastfeeding, that could assist in reaching this goal.

Stunted growth in childhood has both short- and long-term consequences for health, development, and future economic productivity [11]. Our ways of addressing child stunting are not entirely evidence-based—a so-called streetlight effect is apparent. In other words, we focus our nutrition interventions on what is in the light, the stunted child. Feeding programs, water, sanitation and hygiene programs, and other efforts to fight infections are all needed, but have small or no effects on stunting prevalence. We need to redefine our search for solutions where the evidence is: interventions that are effective during or before pregnancy [12,13].

The commercially available versions of ready-to-use therapeutic foods are excellent tools in the management of severe acute malnutrition at hospitals, clinics, and in humanitarian emergencies; and may also be effective for home-based use by routine health services [14]. There are, however, numerous difficulties in achieving satisfactory program effectiveness in community-based management of uncomplicated severe acute malnutrition. In the accompanying Special Issue, a number of the reported research studies illustrate some of these practical problems [15,16].

A substantial body of evidence suggests that poor nutrition before and during pregnancy has long-term consequences for future health. The DOHaD paradigm [17], and the evidence from famine in war and disasters, suggest that an adequate diet before and during pregnancy could reduce the global epidemic of chronic diseases [18]. However, there are very few attempts to develop research and programs addressing the need for improved nutrition in adolescence, before conception, and during pregnancy to promote the future health of offspring [19]. In the Special Issue, Sophie Moore and colleagues add to this knowledge by reporting the effect of prenatal nutrition supplementation and the impact on antibody responses to some child vaccinations [20].

We are in the midst of the “decade of action on nutrition” declared at the World Health Assembly in 2016 [2]. The second Sustainable Development Goal includes targets to end hunger, achieve food security, improve nutrition, and promote sustainable cultivation of crops. This year’s Food Security and Nutrition report included a wide range of indicators—and the challenges demand global and national actions in all sectors [1]. Among these goals, what should the health system aim to deliver? One may question whether nutrition is still an orphan within the global health systems architecture, and there is a need for a continuum of nutrition interventions and care to be recognized, from adolescence, through pregnancy and childbirth, to infancy and into childhood of the next generation. The nutritional needs of adolescent girls need particular emphasis [21].

The global state of maternal and child nutrition shows some progress, but several new challenges are apparent. Nutrition should be promoted throughout the life cycle, but currently we often overlook some of the most critical stages, including adolescence. All sectors of society need to be involved to address these problems, but in the health sector, ownership and accountability are often weak. In the future, population growth and the ongoing climate crisis are expected to intensify food insecurity and increase the risk of undernutrition [22], which could be exacerbated by unpredictable migration patterns and social unrest. As showcased by the new contributions in this *PLOS Medicine* Special Issue, which will be complemented by related studies to be published in *PLOS ONE*, new and creative approaches with a focus on research, program development and implementation are key to improving health outcomes for mothers and babies.

## References

[1] FAO, IFAD, UNICEF, WFP and WHO. The State of Food Security and Nutrition in the World 2019. Safeguarding against economic slowdowns and downturns. Rome, FAO, 2019. https://www.unicef.org/media/55921/file/SOFI-full-report.pdf

[2] Demaio AR, Branca F. Decade of action on nutrition: our window to act on the double burden of malnutrition. BMJ Glob Health. 2018;3(Suppl 1):e000492 10.1136/bmjgh-2017-000492 29379647 PMC5759727

[3] Heslehurst N, Vieira R, Akhter Z, Bailey H, Slack E, Ngongalah L, et al The association between maternal body mass index and child obesity: A systematic review and meta-analysis. PLoS Med. 2019;16(6):e1002817 10.1371/journal.pmed.1002817 31185012 PMC6559702

[4] Chowdhury MAB, Adnan MM, Hassan MZ. Trends, prevalence and risk factors of overweight and obesity among women of reproductive age in Bangladesh: a pooled analysis of five national cross-sectional surveys. BMJ Open. 2018 7 19;8(7): e018468 10.1136/bmjopen-2017-018468 30030307 PMC6059314

[5] Tarry-Adkins JL, Aiken CE, Ozanne SE. Neonatal, infant and childhood growth following metformin versus insulin treatment for gestational diabetes: A systematic review and meta-analysis. PLoS Med. 2019; 16(8): e1002879 10.1371/journal.pmed.1002879 31386659 PMC6684046

[6] Akhter Z, Rankin J, Ceulemans D, Ngongalah L, Ackroyd R, Devlieger R, et al Pregnancy after bariatric surgery and adverse perinatal outcomes: A systematic review and meta-analysis. PLoS Med. 2019; 16(8): e1002866 10.1371/journal.pmed.1002866 31386658 PMC6684044

[7] Lecorguille M, Jacota M, de Lauzon-Guillain B, Forhan A, Cheminat M, Charles MA, et al An association between maternal weight change in the year before pregnancy and infant birth weight: ELFE, a French national birth cohort study. PLoS Med. 2019; 16(8): e1002871 10.1371/journal.pmed.1002871. 31430274 PMC6701747

[8] Chivese T, Norris SA, Levitt NS. Progression to type 2 diabetes mellitus and associated risk factors after hyperglycemia first detected in pregnancy: A cross-sectional study in Cape Town, South Africa. PLoS Med. Forthcoming 2019. 10.1371/journal.pmed.1002865 PMC6733438 31498800

[9] Bhattacherjee NV, Schaeffer LE, Marczak LE, Ross JM, Swartz SJ, Albright J, et al Mapping exclusive breastfeeding in Africa between 2000 and 2017. Nature Med. 2019 10.1038/s41591-019-0525-0 PMC6749549 31332393

[10] Black RE, Victora CG, Walker SP, Bhutta ZA, Christian P, de Onis M, et al Maternal and child undernutrition and overweight in low-income and middle-income countries. Lancet. 2013 8 3;382(9890):427–51. 10.1016/S0140-6736(13)60937-X 23746772

[11] Victora CG, Adair L, Fall C, Hallal PC, Martorell R. Maternal and child undernutrition: consequences for adult health and human capital. Lancet. 2008;371:340–57 10.1016/s0140-6736(07)61692-4 18206223 PMC2258311

[12] Uauy R, Kain J, Corvalan C. How can the Developmental Origins of Health and Disease (DOHaD) hypothesis contribute to improving health in developing countries? Am J Clin Nutr. 2011 12;94(6 Suppl):1759S–1764S. 21543534 10.3945/ajcn.110.000562 PMC3808270

[13] Svefors P, Rahman A, Ekstrom E-C, Khan AI, Lindström E, Persson LÅ, et al Stunted at 10 Years. Linear Growth Trajectories and Stunting from Birth to Pre-Adolescence in a Rural Bangladeshi Cohort. PLoS ONE. 2016;11(3):e0149700 10.1371/journal.pone.0149700 26934484 PMC4775024

[14] Schoonees A, Lombard MJ, Musekiwa A, Nel E, Volmink J. Ready-to-use therapeutic food (RUTF) for home-based nutritional rehabilitation of severe acute malnutrition in children from six months to five years of age. Cochrane Database Syst Rev. 2019 5 15;5(3):CD009000. 31090070 10.1002/14651858.CD009000.pub3 PMC6537457

[15] Huybregts L, Le Port A, Becquey E, Zongrone A, Baba FM, Rawat R, et al Impact on child acute malnutrition of integrating small-quantity lipid-based nutrient supplements into community-level screening for acute malnutrition: A cluster randomized controlled trial in Mali. PLoS Med. 2019; 16(8): e1002892 10.1371/journal.pmed.1002892. PMC6711497 31454356

[16] Kangas ST, Salpeteur C, Nikiema V, Talley L, Ritz C, Friis H, et al Impact of reduced dose of ready-to-use therapeutic foods in children with uncomplicated severe acute malnutrition: A randomised non-inferiority trial in Burkina Faso. PLoS Med. 2019; 16(8): e1002887 10.1371/journal.pmed.1002887. PMC6711495 31454351

[17] Hanson M, Godfrey KM, Lillycrop KA, Burdge GC, Gluckman PD. Developmental plasticity and developmental origins of non-communicable disease: Theoretical considerations and epigenetic mechanisms. Progress in Biophysics and Molecular Biology. 2011 7;106(1):272–80. 10.1016/j.pbiomolbio.2010.12.008 21219925

[18] Roseboom TJ, Painter RC, van Abeelen AFM, Veenendaal MVE, de Rooij SR. Hungry in the womb: what are the consequences? Lessons from the Dutch famine. Maturitas. 2011 10;70(2):141–5. 10.1016/j.maturitas.2011.06.017 21802226

[19] Fleming TP, Watkins AJ, Velazquez MA, Mathers JC, Prentice AM, Stephenson J, et al Origins of lifetime health around the time of conception: causes and consequences. Lancet. 2018 5 5;391(10132):1842–52. 10.1016/S0140-6736(18)30312-X 29673874 PMC5975952

[20] Okala SG, Darboe MK, Sosseh F, Sonko B, Faye-Joof T, Prentice AM, et al Impact of nutritional supplementation during pregnancy on antibody responses to diphtheria-tetanus-pertussis vaccination in infants: A randomized trial in The Gambia. PLoS Med. 2019; 16(8): e1002854 10.1371/journal.pmed.1002854 31386660 PMC6684039

[21] Bhutta ZA, Lassi ZS, Bergeron G, Koletzko B, Salam R, Diaz A, et al Delivering an action agenda for nutrition interventions addressing adolescent girls and young women: priorities for implementation and research. Ann N Y Acad Sci. 2017 4;1393(1):61–71. 10.1111/nyas.13352 28436103

[22] Costello A, Abbas M, Allen A, Ball S, Bell S, Bellamy R, et al Managing the health effects of climate change: Lancet and University College London Institute for Global Health Commission. Lancet. 2009 5 16;373(9676):1693–733. 10.1016/S0140-6736(09)60935-1 19447250

